# The oldest “brown mesophotic” coral-stromatoporoid ecosystem from the Silurian of Gotland was functionally similar to modern turbid reefs

**DOI:** 10.1038/s41598-025-26596-8

**Published:** 2025-11-27

**Authors:** Mikołaj K. Zapalski, Jan J. Król, Piotr Łuczyński, Stanisław Skompski, Błażej Berkowski, Kyle Morgan

**Affiliations:** 1 Faculty of Geology, University of Warsaw, Żwirki i Wigury 93, Warszawa, 02-089 Poland; 2 Institute of Geology, Adam Mickiewicz University, B. Krygowskiego 12, 61‑680 Poznan, Poznań, Poland; 3 Earth Observatory of Singapore, Nanyang Technological University, Nanyang, Singapore; 4 Asian School of the Environment, Nanyang Technological University, Nanyang, Singapore

**Keywords:** Functional ecology, Paleoecology, Turbidity, Sedimentation, Convergence, Ecology, Ecology, Ocean sciences

## Abstract

Coral reefs generally thrive under high light conditions. As light decreases with depth, corals may adapt their morphology to optimise light capture. However, these same changes in morphology (e.g., platy forms) may also occur in response to light attenuation caused by turbidity within shallow waters. In the fossil record the occurrence of turbid shallow-water ecosystems has been largely restricted to the Meso- and Cenozoic. Only a single example of an ecosystem functionally analogous to modern turbid reefs has been identified from the Palaeozoic (Devonian). Here we report a Silurian (~ 425 Ma) reef ecosystem from Gotland, Sweden, composed predominantly of platy tabulate corals. Sedimentological data suggest shallow depths, high sedimentation rates and an unconsolidated substrate. We interpret the Gotland reef to be functionally similar to modern turbid reefs, making it the oldest known turbid reef system, extending records by nearly 40 million years. This suggests that platy growth forms and colonies similar to modern funnel-shaped forms first emerged during the Silurian as a novel strategy that facilitated coral expansion in turbid, low-light environments. The recurrence of these forms across geological time suggests strong functional convergence driven by similar environmental pressures, evolving at least twice in both tabulate and scleractinian corals.

## Introduction

Modern shallow clear-water coral reefs are highly productive marine ecosystems that are widespread within tropical seas. However, these reef types represent only a small proportion of global reef diversity^1^. Other reef ecosystems are both widespread and ecologically important but remain far less understood. For example, mesophotic reefs, which occur at depths of 30–150 m, may be even more extensive than their shallow-water counterparts^1^. Similarly, turbid reefs, defined as coral communities that thrive under reduced water clarity, are abundant within coastal nearshore settings.

Turbidity refers to the amount of light absorbed and scattered by suspended particulate matter (SPM), both organic and inorganic^2^, and is regarded as a major stressor for corals^2,3^. Consequently, modern turbid reefs are considered light-limited coral habitats, usually located in coastal or estuarine settings^3^. They can form in areas affected by wave-driven resuspension, riverine discharge, or strong tidal currents, and are estimated to make up around 12% of the world’s reefs^4^. Despite being regarded as marginal environments for coral growth, naturally turbid reefs are often extensive, resilient, and ecologically significant^5^.

Many corals in turbid settings exhibit platy growth morphologies, which maximize surface area for light capture under reduced irradiance and sustain coral–algal symbiosis^6^. Other growth forms may locally dominate, depending on local conditions, as factors other than light attenuation may play an important role in coral growth strategies. In this respect, turbid reefs share similarities with deeper light-limited mesophotic coral ecosystems and have been termed “brown mesophotic” in contrast to the “blue mesophotic” of deeper and clearer waters^7,8^. Many turbid reef corals demonstrate remarkable resilience, with corals exhibiting diverse adaptations to low-light and high-sediment environments^9–11^. Such resilience suggests that turbid reefs may serve as refugia in a changing climate, making it imperative to understand their ecological and geological history^11^.

Despite their significance, modern turbid reefs are understudied, and their fossil record is fragmentary. From the Mesozoic and Cenozoic, very few well-documented examples are known e.g^12–14^. During the Palaeozoic era, coral reef ecosystems expanded, and in many respects resembled modern reefs^15–18^. However, the Palaeozoic reefs were constructed by extinct coral groups such as tabulates and rugosans, with stromatoporoid sponges also serving as major framework builders, especially in shallow water^19,20^. To date, the only documented example of a Palaeozoic turbid reef is from the Middle Devonian (Givetian) of Queensland^21^. This coral assemblage, dominated by platy and massive tabulate corals shows remarkable morphological similarities to modern turbid reefs from the inshore Great Barrier Reef^21^.

Here, we report a new coral-dominated biostrome from the Silurian (Ludfordian stage, ~ 425 Ma) of Gotland, Sweden. This well-preserved community of platy tabulate corals and stromatoporoids represents the earliest known turbid reef. Functionally, it resembles modern turbid reefs, with morphologies adapted to low light availability. Forming at the onset of the mid-Palaeozoic, this assemblage provides evidence for the early evolution of coral morphologies suited to turbid, light-limited environments. Importantly, it predates the Queensland example by at least 40 million years, extending the record of turbid reef ecosystems deep into the Silurian.

## Palaeogeographical and geological setting

**Fig. 1:**
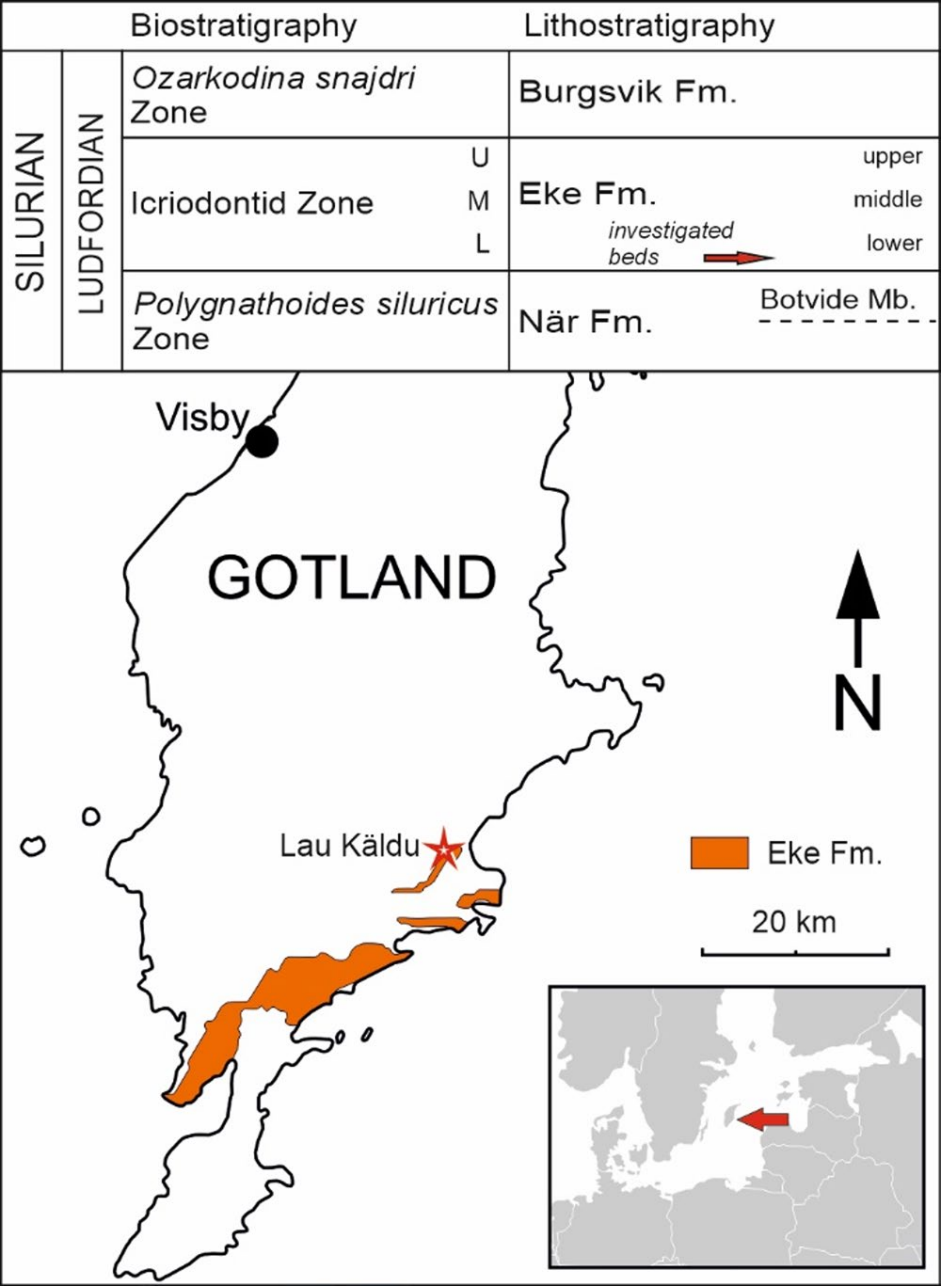
Stratigraphy and locality of the investigated coral biostrome. Stratigraphy after Eriksson and Calner^22^. Location of Lau Käldu after Król et al.^31^.

During the Silurian, the Baltic Basin was located on the tropical shelf of the palaeocontinent Laurussia^22^. The island of Gotland (today located on the Baltic Sea, Sweden) has been known for its Silurian reefs and corals since the XIX century^23–25^. The sedimentary sequence begins with the deep-water Visby Beds (latest Telychian), part of which have been interpreted as representing depths of about 100 m^26^ and regarded as a Mesophotic Coral Ecosystem (MCE)^27^. Evidence suggests that shallow-water reefs may have formed simultaneously with the MCEs in the area^17^, and were similar to those of the overlying shallow-water Högklint Formation^25^. The overall Silurian sedimentary sequence on Gotland records a regressive trend, with several significant hiatuses in reef construction^28^. In the central-southern part of the island, close to its eastern shore, the Eke Formation crops out in several inland exposures (Fig. 1). The beds of this Formation are represented by shallow-water detrital and bioclastic limestones and marls, with occasional small reefal buildups. The Eke Formation is of late Ludfordian age (Icriodontid Conodont Zone), corresponding with the so-called Lau extinction event^28–30^.

This study focuses on a coral-stromatoporoid community within the Eke Formation, exposed as a small outcrop (escarpment) in Lau Käldu (Fig. 1), located 0.65 km ENE of Lau Kyrka in Lau village. The sequence is at least 3 m thick; its base being covered by rubble and vegetation.

## Materials and methods

Fieldwork was conducted in July 2021 and August 2022 on the island of Gotland, Sweden (Fig. 1). The outcrop is located approximately 750 m south from Lau Backar Hill. The site is visible from the main road (road I712) as a small escarpment immediately behind the well of the same name (to the west from the road).

Most of the fieldwork was concentrated on detailed in situ observations of sedimentary characteristics of the investigated biostrome, spatial relationships between bioconstructors and sediment, as well as distribution and shapes of bioconstructors (corals and stromatoporoids). Due to nature protection regulations prohibiting collection of in situ specimens from the outcrops, loose weathered specimens and rock samples were collected from scree deposits, as well as several lithological samples for microfacies studies. In order to ensure that samples come from the investigated bed we have collected specimens only from the surface immediately below the biostrome, and for microfacies sampling we collected rocks that were already detached by weathering, but still in their original position. A total of 172 coral colonies and four samples for microfacies analysis was collected. The majority of coral specimens display good preservation to allow for taxonomic identifications based on their external morphology. Field photographs have been made using Canon EOS 70D camera and 10–18 mm and 24–80 mm lenses. Thin sections were imaged using an Epson Perfection V800 scanner and a Keyence VHX-7000 digital microscope. Figured specimens are housed at the St. Thugutt Geological Museum of the Faculty of Geology, University of Warsaw, Warsaw (abbreviated WG), and at the Institute of Geology, Adam Mickiewicz University, Poznań (abbreviated UAM).

## Results

### Macroscopic field observations

The outcropping limestone succession begins with 2 m of thin-bedded detrital limestones containing crinoids and small rugose corals. These are overlain by a 1 m thick coral-stromatoporoid biostrome, which is the focus of this investigation.

**Fig. 2:**
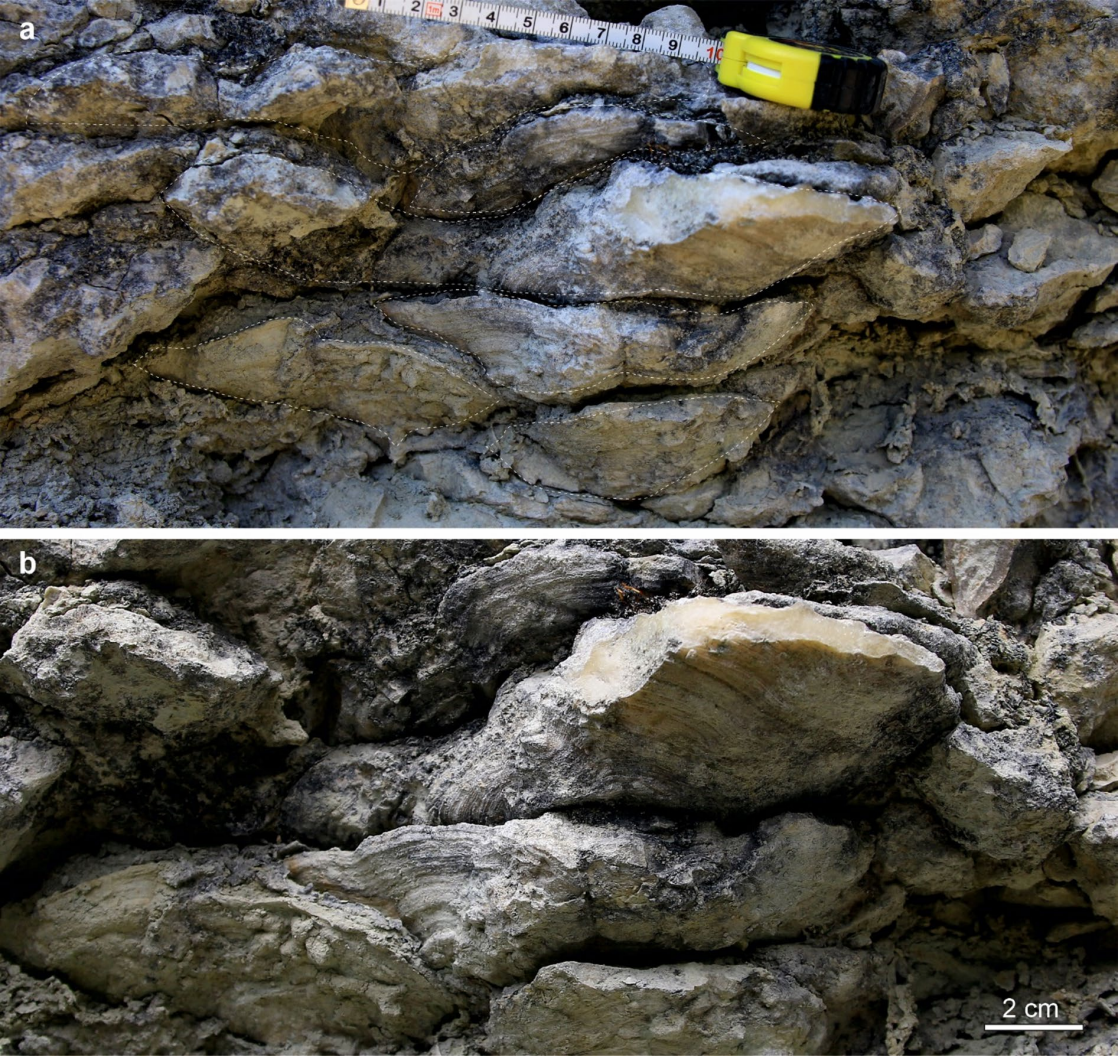
Fragment of biostrome with stacks of platy, tabular and broadly funnel-shaped heliolitids forming framework, in situ, Lau Käldu, Gotland, upper Ludfordian. **a**. General view with coral colony outlines, **b**. Larger view of the central part of a.

The biostrome consists predominantly of densely packed platy tabulate corals preserved in growth position, occasionally forming framework (Fig. 2). Heliolitid corals are the most abundant coral taxa, comprising over 80% of corals exhibiting platy and tabular growth forms^31^. Stromatoporoids, although relatively scarce, are mostly preserved in their original growth position. Stromatoporoids occur with low-profile growth morphologies. Solitary and weakly colonial rugosans, crinoids and branching bryozoans are also present. Notably, the slope of the escarpment changes its inclination immediately above the biostromal unit from very steep to almost flat, suggesting a change in lithology following the reefal sedimentation. This biostrome likely corresponds to the lower part of the Eke Formation (Lower Icriodontid Conodont Subzone) in Eriksson and Calner^22^.

Lateral to the described succession, on the southern side of the escarpment and at approximately the same elevation, a ~ 4 m thick unit of massive, slightly nodular limestone is exposed. This unit is dominated by large, massive stromatoporoids displaying a wide array of morphologies, including high-profile growth forms. Due to the presence of a paved path, it is difficult to determine the nature of the transition between the two facies, or their spatial and temporal relationships. However, the similar elevation of both parts of the outcrop suggests the two facies may be contemporaneous.

### Microfacies

**Fig. 3:**
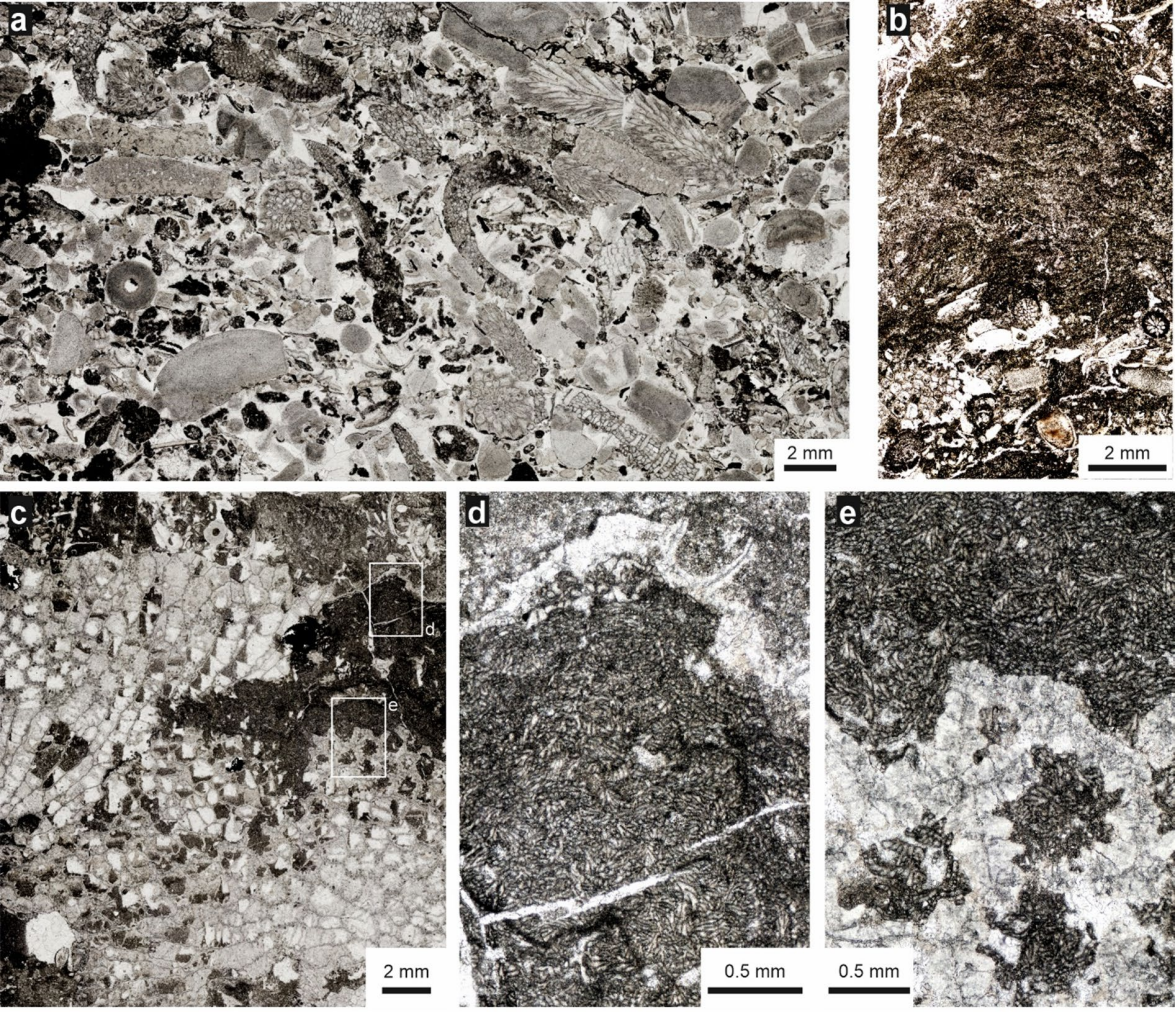
Microfacies of the biostrome at Lau Käldu, Gotland, upper Ludfordian. **a**. Bioclastic grainstone dominated by crushed tabulates and crinoidal debris, with accessory bryozoans. **b**. Spongiostromate (thrombolitic) coats. **c**. Tabulate clast encrusted by thick envelope of a possible alga *Rothpletzella* (**d** and **e**).

In thin section, the detrital and bioclastic limestones of the lower Eke Formation are classified as grainstones to rudstones dominated by crushed tabulate corals with fragments up to 1 cm in length, along with abundant crinoidal debris, dispersed in micritic and fine-grained matrix (Fig. 3). Accessory components include bryozoan fragments, crushed brachiopods, and bioclastic material arranged in a disorganised, poorly sorted fabric.

In contrast, the microfacies of the tabulate-stromatoporoid biostrome differ markedly from the bioclastic grainstones and rudstones. The matrix of the biostrome is dominated by densely packed wackstone to packstone, containing large bioclasts of tabulate corals and stromatoporoids. The skeletal components are frequently encrusted by thick micritic coatings formed by spongiostromate, microbial structures, similar to cyanobacterial in origin (Fig. 3b). Additionally, equally thick encrustations formed by typical specimens of microproblematic *Rothpletzella* were common and deeply penetrated the structure of tabular bioclasts (Fig. 3b–d).

### Corals

Rugose corals are relatively uncommon in the studied beds and are represented by solitary and rare weakly colonial (fasciculate) corals. Among solitary taxa, the most common are relatively small cystiphyllimorphs belonging to tryplasmatids (*Stortophyllum* sp.) and cystiphyllids (*Hedstroemophyllum* sp.), and less frequent tabular ketophyllids (*Dokophyllum* sp.). The only weakly colonial forms are the phaceloid cistimorph *Microplasma* sp. and arachnophyllid *Entelophyllum fasciculatum*, which also occur as solitary. Both solitary and colonial rugosans are small (corallite diameters of ~ 0.5 to 1.5 cm), typically elongate, and show evidence of frequent rejuvenations. In their basal portions, corallites developed rhizoid structures that attach to larger skeletal elements, primarily the skeletons of other corals.

**Fig. 4:**
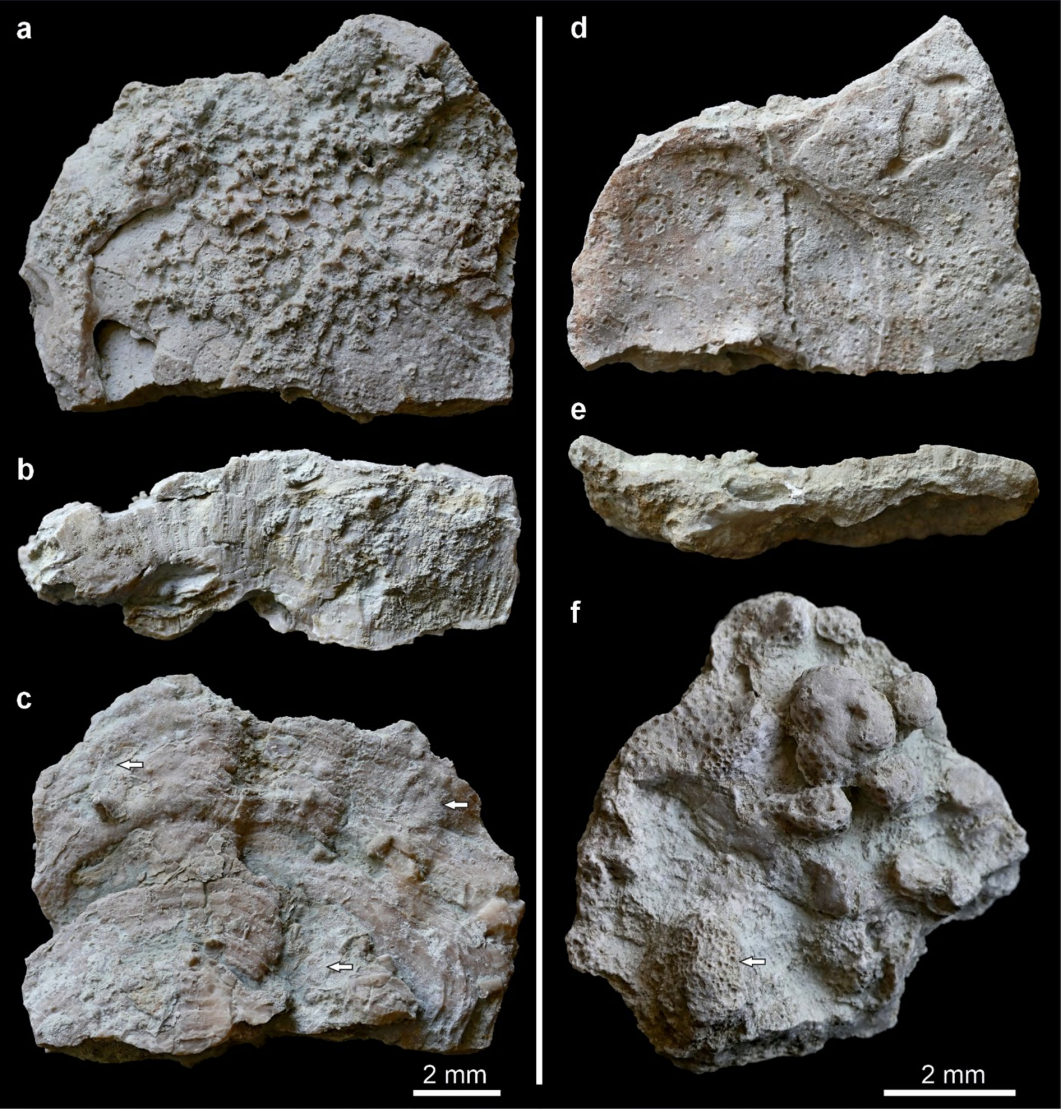
Platy heliolitids from Lau Käldu, Gotland, upper Ludfordian. a–c: A fragment of a platy corallum of *Stelliporella parvistella*. a - top view with abundant *Aulopora* sp. sclerobionts; b - side view; c - bottom view with visible growth increments and bryozoan sclerobionts (arrows). d–e: A fragment of a platy corallum of *Heliolites interstinctus*. d - top view; e - side view. f: An irregular, “rising” corallum (*sensu* Król et al. 2024^31^ of *S. parvistella*; top view. Favositid sclerobiont visible in bottom left (arrow).

The reef-building taxa are exclusively tabulate corals, represented by two heliolitid families (Heliolitidae and Stelliporellidae) and two favositid families (Favositidae and Alveolitidae). Most of the specimens investigated form platy colonies, sensu Rosen et al.^6^, with height-to-width ratio less than 1:4 (Fig. 4). Some platy coral colonies, as observed in growth position, are arranged in broadly funnel-shaped stacks, but with slightly concave upper surfaces. The second largest morphological group are tabular colonies with proportions between 1:3 and 1:4^31^. Colonies are mostly fragmentary, collected from the rubble (scree), where the largest fragments reach 26 cm in diameter. Based on holothecal growth rings, complete colonies may have reached twice that size in diameter. Colonies reach up to 5.3 cm in thickness.

**Fig. 5:**
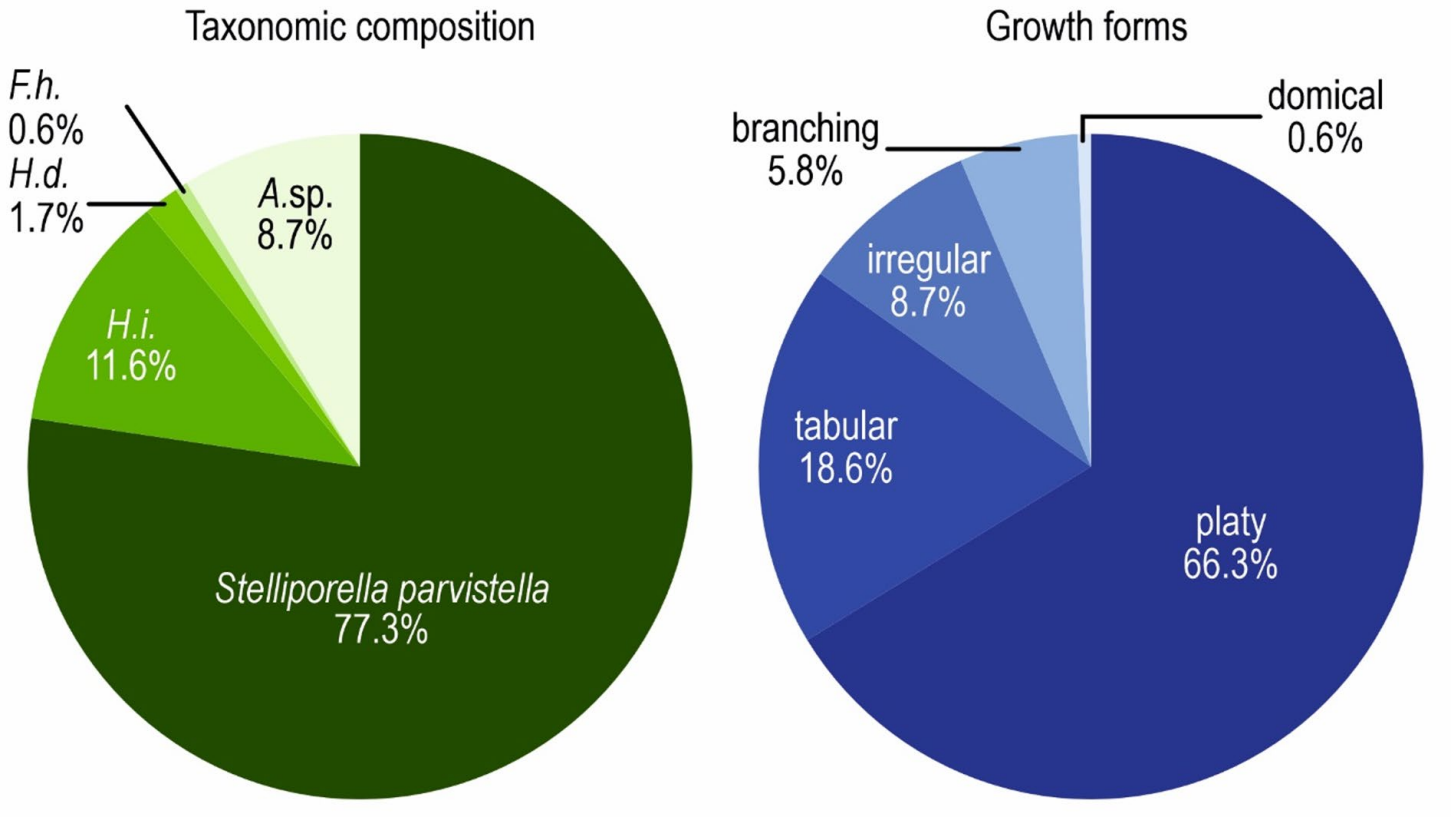
Taxonomic composition and growth forms of reef-building tabulate corals from Lau Käldu. *H.i. – Heliolites interstinctus*,* H.d. – Heliolites daintreei*,* F.h. – Favosites hisingeri*,* A*.sp. – *Alveolites* sp. *N* = 172.

Heliolitids are the most abundant group in the biostromal assemblage (over 90%), but are characterised by very low taxonomic diversity, with only three species present (Fig. 5). Of all collected reef-building (non-auloporid) tabulate coral specimens (172 in total), 85% of specimens are platy or tabular. The dominant species is *Stelliporella parvistella* (77% of specimens), comprising both platy (61% of *S. parvistella* specimens) and tabular (20%) colonies, as well as the less common irregular (11%) and branching (7%) forms. Both platy and tabular forms include broadly funnel-shaped forms, visible on Fig. 2. Notably, 30% of *S. parvistella* specimens exhibit vertical protrusions, finger-like or resembling tumours (Fig. 4f). Colonies of *S. parvistella* classified as “irregular” are generally platy or tabular forms with such protrusions developing on their upper surfaces. The second most abundant heliolitid species (11.6%, 20 specimens) is *Heliolites interstinctus*, with almost exclusively platy (70%) and tabular (25%) growth forms. *H. daintreei* was rare, comprising only 1.7% (3 specimens) of the heliolitid assemblage, and represented exclusively by platy forms. An additional 8.7% (15 specimens) of the tabulate assemblage consists of an alveolitid tentatively identified as *Alveolites* sp. 1, which likely represents a new species. Favositids are rare and represented by a single colony of *Favosites hisingeri.* The overall distribution of tabulate growth forms is presented in Fig. 5.

**Fig. 6:**
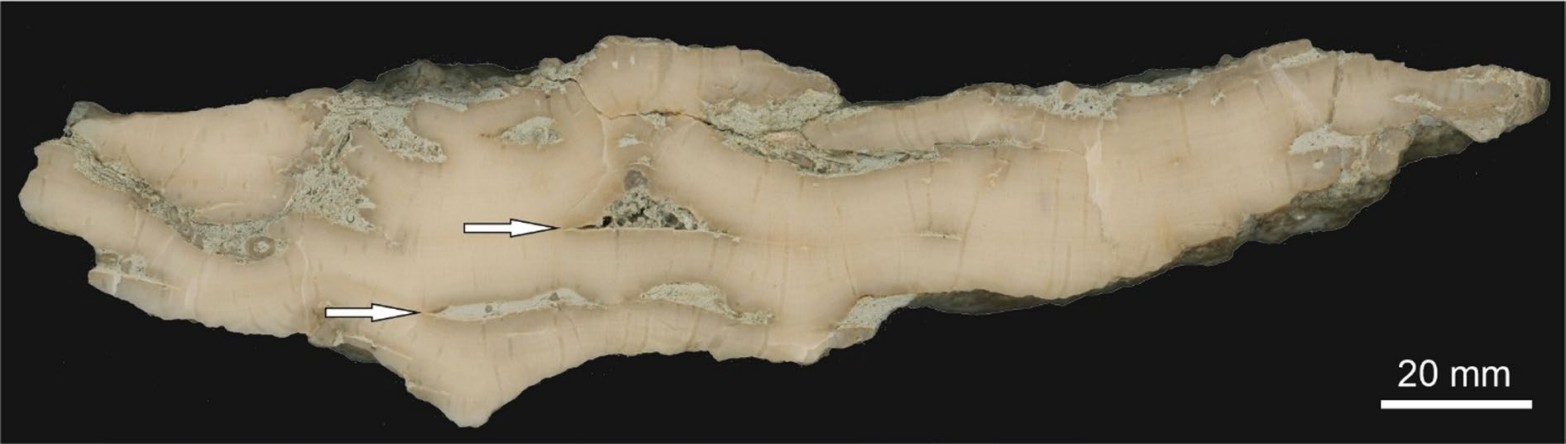
*Stelliporella parvistella* from Lau Käldu, Gotland, upper Ludfordian, longitudinal section through platy colony (corallum), with interpreted sediment influx events (arrows show examples). Note the slightly convex top surface of the colony.

One colony of *S. parvistella* was longitudinally sectioned to examine growth architecture. The specimen, about 20 cm in diameter, revealed several growth-interruption surfaces, some marked by visible sediment influx (Fig. 6), as observed in acid-etched section. Accessory tabulate faunas are represented by two species of encrusting auloporids and rare branching pachyporids (*Striatopora* sp.).

### Stromatoporoids

In the biostrome, the stromatoporoids are relatively scarce and greatly outnumbered by tabulate corals. They are represented solely by laminar and low domical forms ^see 32,33^ for definitions and morphometrical features]. All specimens were found in normal orientation, with no evidence of overturning, indicating preservation in growth position. These stromatoporoids are moderate in size, with basal lengths not exceeding 10 to 12 cm, and vertical heights between 1 and 4 cm. Both lower and upper surfaces are flat, with no vertical raggedness observed. Where visible, latilaminae arrangements are in an enveloping pattern.

In contrast, stromatoporoids from the adjacent massive nodular limestones are the dominant macrofossils and exhibit a wider range of morphometrical features. These include several complex morphotypes, such as high domical, extended domical and low bulbous types, with both enveloping and non-enveloping latilaminae arrangements. Specimens in this unit are relatively large, often exceeding 20 cm in diameter and are also in situ.

### Other fauna

Other associated fauna includes abundant crinoid skeletal elements, bryozoans, and rare brachiopods, predominately orthids. Coral colonies are frequently encrusted by bryozoans and microconchids, indicating prolonged exposure on the seafloor. Epizoans such as bryozoans and auloporids occasionally occur on the undersides of coral colonies.

## Discussion

The investigated biostrome is situated within the basal part of the Eke Formation, which has been interpreted as representing a relatively calm and shallow environment, based on the prevalence of cauliflower-like oncoids^30^. Typically, oncoids are spherical due to constant tumbling on the seafloor. However, in cauliflower-like specimens, growth is irregular due to less frequent overturning. Evidence of subaerial exposure, including karst phenomena^34^ and the presence of *Palaeomicrocodium*, further support deposition in very shallow environments near the water-air interface^30^. In general, the Formation has been interpreted as representing subtidal sediments deposited on the seaward margin of the carbonate platform^29^.

Our microfacies analysis of the biostrome suggests at least occasional high energy episodes, as indicated by fragmented bioclasts, and sediment infill preserved within coral colonies along with sediment-induced, growth interruptions (Fig. 6). The chaotic orientation of bioclasts, and their poor sorting, indicates high-energy deposition typical of shallow-water environments and high sedimentation rates. The relative scarcity of encrusting fauna on the undersides of coral colonies suggests sediment accumulation that inhibited biofouling. The undersides of colonies are generally bare, but sparsely colonised by epizoans such as bryozoans and auloporids. On the other hand, their upper surfaces are often overgrown by auloporid tabulates (Fig. 4a). In contrast, corals from the Visby Beds commonly have encrusted undersides, indicating potentially lower sedimentation rates and a relatively clear-water setting^6^.

An important new line of evidence in support of a shallow-water environment is the abundance of *Rothpletzella* encrustations (Fig. 3) within the biostromal facies. These fossils are interpreted as belonging to the green algal order Bryopsidales^35^, which suggests a phototrophic mode of feeding and, consequently, the preference of *Rothpletzella* for shallow water environment. This observation is consistent with generally shallow-water interpretations of the occurrence of thrombolitic structures^36^.

Our findings therefore support previous interpretations^29,30^ of the Eke Formation sedimentary environment. The depositional environment was characterised by shallow depths, high sedimentation rates and an unconsolidated limestone mud substrate. Any water movement, such as wave action or currents, resulted therefore in increased turbidity and decreased irradiance.

Morphological features of stromatoporoid skeletons are widely recognised as reliable environmental indicators, allowing reconstructions of key sedimentary features of their habitats e.g^37^^38^. In turbid environments several environmental factors likely influenced the stromatoporoid morphologies. These factors include light availability, substrate type and consolidation, and the occurrence of fine sediment particles suspended in the water column.

The extent to which stromatoporoids were photosensitive is still debated, though several features support this interpretation: for example, changes of growth axis towards vertical of specimens inhabiting inclined surfaces^38^ and rapid growth in shallow habitats^39,40^. Stearn^41^ proposed that stromatoporoids were likely mixotrophs, with metabolism partially supported by photosynthesis. If the above is correct, we can infer that the light limitation was probably the cause of the relatively small sizes of the specimens, while their flat morphology can be interpreted as an adaptation to soft, muddy bottom substrates^42^.

However, a potentially significant environmental factor limiting stromatoporoid growth in the brown mesophotic setting, eventually causing local extinction of stromatoporoids and driving a transition to larger and higher profile forms was pore clogging. As filter feeders, stromatoporoids – which are similar to modern sponges – were highly susceptible to sediment clogging of their canal systems^43^. Recurring turbidity episodes caused by the resuspension of seafloor sediment likely obstructed filtration, leading to reduced growth and mortality. This resulted in flat laminar forms, with only single or few latilaminae (major growth bands).

The stromatoporoid assemblage from the massive nodular limestones, exposed laterally to the biostrome, distinctly differs in morphology and thus also in the resulting environmental interpretation. The stromatoporoids occur in a wide array of morphologies, and in some cases are dominated by high profile forms, such as high- and extended domical and bulbous. The diversity of initial attachment surfaces, latilaminae arrangements, and upper surfaces suggest a “healthy” and well-developed stromatoporoid community typical of a clear-water shallow marine setting, with dynamic sedimentation punctuated by high energy episodes. This facies may represent a palaeotopographical high point within the sedimentary basin, elevated above the adjacent low-lying muddy seafloor, where the mesophotic, turbid-water biostrome developed.

The morphological features of rugose corals are consistent with a turbid water interpretation. The dominance of solitary cystiphyllid rugose corals with such anatomical features as well-developed transseptal, cystiphylloid dissepimentaria and attachment structures (i.e., talons and rhizoid structures), as well as frequent rejuvenations indicate that the environment was dynamic and turbid^44–46^. Rare colonial rugosans are exclusively represented by fasciculate (phaceloid) colonies, a growth form that is associated with relatively high rates of sedimentation^47^. These corals have loosely packed, elongate corallites that grew partly embedded in the soft sediment, allowing them to grow much faster than massive colonies. This “mud-sticker” growth strategy appears advantageous in the muddy environments with higher sedimentation rates^48,49^.

It seems that the platy growth strategy developed in the early Wenlock (at the latest), as an adaptation to depleted light levels^27^. Somewhat younger heliolitid corals from the Lau Käldu biostrome exhibit predominantly platy and tabular growth forms, and are dominated by *Stelliporella parvistella*, a species which is known for its high morphological plasticity^31,50^. Platy morphologies can either be advantageous on soft substrates^51^, for the competition for space and nutrients on reefs, or, in photosymbiotic corals, as an adaptation to low light conditions^50,52^. However, *S. parvistella* has also been reported on soft-bottom environments, such as the Wenlock Blåhäll biostrome on Gotland, where it formed branching rather than platy colonies^50,53^. Similarly, Devonian heliolitids on soft-substrates and on reefs characterised by intense space competition also did not form platy colonies^54,55^. We can therefore infer that the dominance of platy and tabular growth forms of heliolitids from Lau Käldu is likely an adaptation to low light conditions^31^. This is further supported by the presence of “rising” platy, tabular and irregular heliolitids with vertical surface protrusions, strongly resembling *Porites sillimaniani* from modern reefs^56^, a species known to be phenotypically very plastic, and forming finger-like outgrowths in turbid environments.

Heliolitids formed plocoid colonies that were relatively highly integrated in comparison to most other Palaeozoic corals^31^, a trait that is commonly associated with photosymbiotic corals^6,57,58^. Heliolitids also displayed a capacity for colony-wide responses to localised stress, including growth interruptions and regenerative healing of lesions, further indicative of high colony integration^31,54,55,59^. One such growth-interruption surface is illustrated in Fig. 6 . Król et al.^31^ also noted that, unlike many other tabulate groups, heliolitids were restricted to relatively shallow water environments in the photic zone, and showed light-induced skeletal plasticity, particularly within the Visby Formation on Gotland. These traits suggest that at least some heliolitid taxa, including *Stelliporella parvistella*, were likely photosymbiotic.

Zapalski et al.^21^ described a coral community from the Givetian of Queensland (Fanning River site), interpreted as having developed in very shallow water under high turbidity. This assemblage was dominated by platy coenitids and alveolitids, with abundant heliolitids, but forming massive colonies. In contrast, the Silurian biostrome described here is taxonomically and functionally distinct, with heliolitids visibly dominant among the platy growth forms. Such divergence might be caused by differences in ecological preferences of given taxa, varying degrees of genetic control over skeletal architecture in different heliolitid taxa^50,60^ or could signal a general shift in phenotypic response to increased sediment in the water column, suggesting the emergence of a novel morphological strategy of coping with sediment inputs. The Fanning River coral assemblage is also characterised by the presence of broadly funnel-shaped colonies, somewhat similar to these in the Lau Käldu biostrome. Riegl^61^ demonstrated that funnel-shaped colonies may be an adaptation to turbid environments, potentially increasing sediment removal from the colony and/or increasing polyp survival at the edges of colonies. While our specimens (Figs. 2 and 6) are much broader and flatter than the colonies discussed by Riegl et al.^61^, we can speculate that such a shape already played some role in sediment removal.

Although the Silurian MCEs described here pre-date the emergence of scleractinian coral reefs by over 200 million years, they exhibit apparent functional and morphological similarities to modern turbid reef systems. Modern turbid coral reefs typically develop in shallow (< 10 m) coastal and shelf settings, where light availability is limited by elevated inputs of terrestrial sediments and organic matter^62,63^. Coral communities are often dominated by platy and massive coral morphologies, with branching corals being less common and generally restricted to very shallow areas in the upper photic zone^10,64^. These consistent associations imply that such morphotypes provide a competitive advantage in surviving the environmental perturbations typical of turbid settings. These patterns in modern turbid coral morphologies reflect those in the Silurian biostrome investigated here, despite the great difference in age and taxonomy.

Turbidity and reduced light availability strongly influence coral morphology and reef-building strategies. In modern environments, platy corals often grow as thin horizontal plates to maximize light capture and shed sediments in high-flow settings^65^. At Paluma Shoals (GBR)^64^ and reefs offshore Singapore^66^, foliose *Turbinaria* spp. colonies have also been shown to form funnel-shaped stacks, as an adaptation to chronic sedimentation and low light, similar to those seen in the Silurian biostrome. Vertical, finger-like protrusions, like those observed in *Stelliporella parvistella*, are also frequently observed on the upper surfaces of turbid platy corals (e.g., *Montipora*, *Porites*), and can also create clubbed branches (e.g., *Merulina*). While not well documented, these altered morphologies are associated with sediment shedding and increased light capture^67^, or interactions with coral-associated invertebrates (e.g., barnacles, worms, crustaceans^68^. Episodic sedimentation-driven mortality and regrowth of coral tissue, which incorporates sediment into skeletal structures^69^, is another shared feature of both fossil and modern turbid reef systems. The consistent presence of these morphological adaptations across time highlights an important convergent response of corals from different lineages to turbid conditions.

Similarities also extend to the geomorphic development of reef structures. Similarly to the Silurian reef described here, Holocene reef cores from the Great Barrier Reef^64,70^ and Southeast Asia^71,72^ show that turbid coral assemblages have persisted throughout the mid- to late-Holocene, often initiating on consolidated antecedent substrates with small, solitary coral colonies. Although early colonizers grow slowly, they form the foundation for subsequent reef accretion. In modern reefs, as reef elevation increases, improved light availability and sediment winnowing by tidal currents facilitate the transition to more complex morphologies, such as tabular, branching, and massive forms^9,73^, as well as carbonate-dominated sediments^74,75^. If this model is applied to the Silurian biostrome, the presence of poorly sorted bioclasts and lithoclasts suggests deposition in a high-energy, shallow-water setting, influenced by tidal or wave-induced turbulence. Such environments are inconsistent with low-energy, deep mesophotic settings and support the interpretation that light limitation in this case was driven by turbidity, not depth. Identifying such analogues provides valuable context for reconstructing ancient mesophotic environments and understanding the environmental controls that have shaped reef ecosystems through deep time.

## Conclusions

1. The Silurian (Ludfordian, ~ 425 Ma) community from Gotland dominated by platy tabulate corals developed under shallow-water, turbid conditions and can be classified as a the oldest known to date “brown” mesophotic coral ecosystem.
2. Platy growth in corals, as an adaptation to depleted light and turbid environments, appeared as early as the Ludfordian. Broadly funnel-shaped colonies, likely adapted to high sedimentation rates, also appeared as the Ludfordian. Therefore, such a strategy evolved at least twice, in divergent groups of corals (scleractinians and tabulates). Such novel adaptations may have contributed to the broad distribution of tabulate corals during the mid-Palaeozoic reef acme.
3. In contrast to typical shallow-water Palaeozoic biostromal accumulations, the studied biostrome was dominated by tabulate corals, not stromatoporoids. We attribute the relative scarcity of stromatoporoids to clogging of pores by fine sediment particles in the turbid brown mesophotic environment.

## Data Availability

All data generated or analysed during this study are included in this published article.

## References

[1] Bongaerts, P. Mesophotic coral ecosystems. *Curr. Biol.* **32**, R345–R346 (2022). 35472416 10.1016/j.cub.2022.03.036

[2] Flores, F. et al. Chronic exposure of corals to fine sediments: lethal and sub-lethal impacts. *PloS ONE*. **7** (5), e37795 (2012). 22662225 10.1371/journal.pone.0037795 PMC3360596

[3] Zweifler, A., O’Leary, M., Morgan, K. & Browne, N. K. Turbid coral reefs: past, present and future—a review. *Diversity* **13**, 251 (2021).

[4] Sully, S. & van Woesik, R. Turbid reefs moderate coral bleaching under climate-related temperature stress. *Glob Chang. Biol.* **26**, 1367–1373 (2020). 31912964 10.1111/gcb.14948 PMC7079097

[5] Browne, N. K., Smithers, S. G. & Perry, C. T. Coral reefs of the turbid inner-shelf of the Great Barrier Reef, Australia: an environmental and geomorphic perspective on their occurrence, composition and growth. *Earth-Sci. Rev.* **115**, 1–20 (2012).

[6] Rosen, B. R. et al. Platy coral assemblages: 200 million years of functional stability in response to the limiting effects of light and turbidity. In *Proc. 8th Int. Coral Reef Symp.* 1, 255–264 (2002).

[7] Renema, W. Large benthic foraminifera in low-light environments. In *Mesophotic Coral Ecosystems* 553–561 (2019).

[8] Majchrzyk, A., Jakubowicz, M., Berkowski, B., Bongaerts, P. & Zapalski, M. K. In the shadow of a giant reef: palaeoecology of mesophotic coral communities from the Givetian of Anti-Atlas (Morocco). *Palaeogeogr Palaeoclimatol Palaeoecol*. **602**, 111177 (2022).

[9] Morgan, K. M., Moynihan, M. A., Sanvlani, N. & Switzer, A. D. Light limitation and depth-variable sedimentation drives vertical reef compression on turbid coral reefs. *Front. Mar. Sci.* **7**, 571256 (2020).

[10] Morgan, K. M., Perry, C. T., Smithers, S. G., Johnson, J. A. & Daniell, J. J. Evidence of extensive reef development and high coral cover in nearshore environments: implications for understanding coral adaptation in turbid settings. *Sci. Rep.* **6**, 29616 (2016). 27432782 10.1038/srep29616 PMC4949480

[11] van Woesik, R. Paleo reefs provide clues for contemporary climate-change refugia. *Cell. Rep. Sustain.* **2**, 100289 (2025).

[12] Kołodziej, B., Salamon, K., Morycowa, E., Szulc, J. & Łabaj, M. A. Platy corals from the middle triassic of Upper Silesia, Poland: implications for photosymbiosis in the first scleractinians. *Palaeogeogr Palaeoclimatol Palaeoecol*. **490**, 533–545 (2018).

[13] Król, J. J., Kołodziej, B. & Bucur, I. I. Coral reefs near the Eocene–Oligocene boundary in the northern Transylvanian Basin, Romania: composition and paleoenvironmental interpretation. *Geol. J.* **53**, 565–579 (2018).

[14] Santodomingo, N., Renema, W. & Johnson, K. G. Understanding the murky history of the Coral Triangle: Miocene corals and reef habitats in East Kalimantan (Indonesia). *Coral Reefs*. **35**, 765–781 (2016).

[15] Zapalski, M. K., Berkowski, B., Skompski, S., Pickett, J. W. & Young, G. C. Ancient depths: unprecedented completeness of mesophotic fish-coral ecosystem from the Devonian of Eastern Gondwana. *Gondwana Res.* **142**, 252–261 (2025).

[16] Majchrzyk, A., Jakubowicz, M., Berkowski, B, Król, J., Zatoń, M., Zapalski, M . Modern-type reef in ancient time – Palaeoecology of a Middle Devonian coral community from Madène El Mrakib (Anti-Atlas, Morocco). *Palaeogeogr Palaeoclimatol Palaeoecol*. **633**, 111876 (2024).

[17] Łuczyński, P., Skompski, S. & Zapalski, M. K. Mesophotic vs. shallow water reefs: ecosystem connectivity in the Silurian of Gotland. *Coral Reefs*. **42**, 1147–1161 (2023).

[18] Zapalski, M. K. Evidence of photosymbiosis in Palaeozoic tabulate corals. *Proc. R. Soc. B.* 281(1775), 20132663 (2014). 10.1098/rspb.2013.2663 PMC3866410 24307674

[19] Wood, R. *Reef Evolution* (Oxford Univ. Press, 1999).

[20] Embry, A. F. & Klovan, J. E. Absolute water depth limits of Late Devonian paleoecological zones. *Geol. Rundsch*. **61**, 672–686 (1972).

[21] Zapalski, M. K., Baird, A. H., Bridge, T., Jakubowicz, M. & Daniell, J. Unusual shallow water Devonian coral community from Queensland and its recent analogues from the inshore Great Barrier Reef. *Coral Reefs*. **40**, 417–431 (2021).

[22] Eriksson, M. J. & Calner, M. A sequence stratigraphical model for the late Ludfordian (Silurian) of Gotland, Sweden: implications for timing between changes in sea level, palaeoecology, and the global carbon cycle. *Facies* **54**, 253–276 (2008).

[23] Murchison, R. On the Silurian and associated rocks in Dalecarlia and on the succession from Lower to Upper Silurian in Smoland, Oland and Gothland, and in Scania. *Trans. Geol. Soc. Lond.* **2**, 652–671 (1846).

[24] Wedekind, R. Die Zoantharia rugosa von Gotland (bes. Nordgotland), Nebst bemerkungen zur biostratigraphie des Gotlandium. *Sveriges Geol. Undersökning*. **19**, 1–94 (1927).

[25] Manten, A. A. *Silurian Reefs of Gotland* Vol. 13 (Elsevier, 1971).

[26] Riding, R. Calcareous algae. In: Jaanusson, V., Laufeld, S. & Skoglund, R. (eds). Lower Wenlock Faunal and Floral Dynamics – Vattenfallet Section, Gotland.*Sveriges Geol. Undersökning C* **762**, 54–60 (1979).

[27] Zapalski, M. K. & Berkowski, B. The Silurian mesophotic coral ecosystems: 430 million years of photosymbiosis. *Coral Reefs*. **38**, 137–147 (2019).

[28] Samtleben, C., Munnecke, A., Bickert, T. & Pätzold, J. The Silurian of Gotland (Sweden): facies interpretation based on stable isotopes in brachiopod shells. *Geol. Rundsch*. **85**, 278–292 (1996).

[29] Calner, M. Silurian carbonate platforms and extinction events—ecosystem changes exemplified from Gotland. *Swed. Facies*. **51**, 584–591 (2005).

[30] Claussen, A. L. & Munnecke, A. Benthic response to the strong Silurian climatic fluctuations—implications from Gotland (Sweden). *Facies* **70**, 14 (2024).

[31] Król, J. J., Berkowski, B., Denayer, J. & Zapalski, M. K. Deducing photosymbiosis in extinct heliolitid corals. *Coral Reefs*. **43**, 91–105 (2024).

[32] Łuczyński, P. Improving the parameterization of stromatoporoid shapes – a detailed approach to stromatoporoid morphometry. *Lethaia* **38**, 143–154 (2005).

[33] Łuczyński, P. Tsunamites versus tempestites: various types of redeposited stromatoporoid beds in the Devonian of the Holy Cross Mountains (Poland), a case study from the Ołowianka quarry. *PLoS ONE*. **17**, e0268349 (2022). 35580129 10.1371/journal.pone.0268349 PMC9113602

[34] Cherns, L. Palaeokarst, tidal erosion surfaces and stromatolites in the Silurian Eke Formation of Gotland, Sweden. *Sedimentology* **29**, 819–833 (1982).

[35] Zatoń, M. & Jarochowska, E. Enigmatic encrusting fossils from the Upper Devonian of Russia: probable *Rothpletzella microproblematica* preserved in three dimensions. *Hist. Biol.* **32**, 837–847 (2020).

[36] Kahle, C. F. Biosedimentology of a Silurian thrombolite reef with meter-scale growth framework cavities. *J. Sed Res.* **A71**, 410–422 (2001).

[37] Łuczyński, P. Stromatoporoid shape and burial ratio changes during growth history and their methodological consequences in morphometrical analyses. *Lethaia* **39**, 339–358 (2006).

[38] Łuczyński, P. Stromatoporoid growth orientation as a tool in palaeotopography: a case study from the Kadzielnia Quarry, Holy Cross Mountains, central Poland. *Acta Geol. Polon*. **59**, 319–340 (2009).

[39] Brunton, F. R. & Dixon, O. A. Siliceous sponge-microbe biotic associations and their recurrence through the Phanerozoic as reef mound constructors. *Palaios* **9**, 370–387 (1994).

[40] Kershaw, S. & Brunton, F. R. Palaeozoic stromatoporoid taphonomy: Ecologic and environmental significance. *Palaeogeogr Palaeoclimatol Palaeoecol*. **149**, 313–328 (1999).

[41] Stearn, C. Functional morphology of the Paleozoic stromatoporoid skeleton. *Treatise Online*. **8**, 1–19 (2010).

[42] Kershaw, S., Wood, R. & Guo, L. Stromatoporoids response to muddy substrates in the Silurian. *GFF* **128**, 131–138 (2006).

[43] Kershaw, S. Treatise Online, 31: part E, Revised, 4, Chap. 13: Paleoecology of the Paleozoic Stromatoporoidea. *Treatise Online*. 10.17161/to.v0i0.3982 (2012).

[44] Sorauf, J. E. The function of dissepiments and marginaria in the Rugosa (Cnidaria, Zoantharia). *Verh Österr Akad. Wiss*. **17**, 11–30 (2007).

[45] Berkowski, B. Life strategies and function of dissepiments in rugose coral *Catactotoechus instabilis* from the Lower Devonian of Morocco. *Acta Palaeontol. Pol.* **57**, 391–400 (2012).

[46] Berkowski, B. & Zapalski, M. K. Large dwellers of the Silurian *Halysites* biostrome: rhizosessile life strategies of cystiphyllid rugose corals from the Llandovery of Gotland. *Lethaia* **51**, 581–595 (2018).

[47] Roniewicz, E. & Stolarski, J. Evolutionary trends in the epithecate scleractinian corals. *Acta Palaeontol. Pol.* **44**, 131–166 (1999).

[48] Rodríguez, S. & Somerville, I. D. Appearance of fasciculate rugose corals in the Viséan and Serpukhovian: a review. *Palaeoworld* **19**, 306–315 (2010).

[49] Echevarría, J., Harguindeguy, F. M., Manceñido, M. O., Carignano, A. P. & Damborenea, S. E. Early Jurassic coral reef development outside Tethys: an example from Western Argentina. *Lethaia* **57**, 1–27 (2024).

[50] Young, G. A. & Scrutton, C. T. Growth form in Silurian heliolitid corals: the influence of genetics and environment. *Paleobiology* **17**, 369–387 (1991).

[51] Insalaco, E. Upper Jurassic microsolenid biostromes of northern and central Europe: facies and depositional environment. *Palaeogeogr Palaeoclimatol Palaeoecol*. **121**, 169–194 (1996).

[52] Gibson, M. A. & Broadhead, T. W. Species-specific growth responses of favositid corals to soft-bottom substrates. *Lethaia* **22**, 287–299 (1989).

[53] Calner, M., Sandström, O. & Mõtus, M-A. Significance of a *Halysitid-Heliolitid* mud-facies autobiostrome from the Middle Silurian of Gotland, Sweden. *Palaios* 15, 511–523 (2000). (2000).

[54] Król, J. J., Denayer, J., Wolniewicz, P. & Zapalski, M. K. Heliolitid corals and their competitors: a case study from the Wellin patch reefs, Middle Devonian, Belgium. *Lethaia*. **54**, 540–557 (2021).

[55] Król, J. J., Jakubowicz, M., Zapalski, M. K. & Berkowski, B. Massive tabulates in competition for space: a case study from Aferdou El Mrakib (Middle Devonian, Anti-Atlas, Morocco). *Palaeogeogr Palaeoclimatol Palaeoecol*. **497**, 105–116 (2018).

[56] Muko, S., Kawasaki, K., Sakai, K., Takasu, F. & Shigesada, N. Morphological plasticity in the coral *Porites sillimaniani* and its adaptive significance. *Bull. Mar. Sci.* **66**, 225–239 (2000).

[57] Coates, A. G. & Jackson, J. B. Clonal growth, algal symbiosis, and reef formation by corals. *Paleobiology* **13**, 363–378 (1987).

[58] Barbeitos, M. S., Romano, S. L. & Lasker, H. R. Repeated loss of coloniality and symbiosis in scleractinian corals. *Proc. Natl. Acad. Sci. USA* 107, 11877–11882 (2010). 10.1073/pnas.0914380107 PMC2900674 20547851

[59] Oren, U., Benayahu, Y., Lubinevsky, H. & Loya, Y. Colony integration during regeneration in the stony coral *Favia favus*. *Ecology* **82**, 802–813 (2001).

[60] Król, J. J. The significance of corallite spacing in heliolitid corals. *Span. J. Paleontol.* **40**10.7203/sjp.31744 (2025).

[61] Riegl, B., Heine, C. & Branch, G. M. Function of funnel-shaped coral growth in a high-sedimentation environment. *Mar. Ecol. Prog Ser.* **145**, 87–93 (1996).

[62] Fabricius, K. E. Effects of terrestrial runoff on the ecology of corals and coral reefs: review and synthesis. *Mar. Pollut Bull.* **50**, 125–146 (2005). 15737355 10.1016/j.marpolbul.2004.11.028

[63] Burt, J.A., Camp, E.F., Enochs, I.C. et al. Insights from extreme coral reefs in a changing world. *Coral Reefs* **39**, 495–507 10.1007/s00338-020-01966-y (2020).

[64] Morgan, K. M., Perry, C. T., Smithers, S. G., Johnson, J. A. & Gulliver, P. Transitions in coral reef accretion rates linked to intrinsic ecological shifts on turbid-zone nearshore reefs. *Geology* **44**, 995–998 (2016).

[65] Todd, P. A., Sanderson, P. G. & Chou, L. M. Morphological variation in the polyps of the scleractinian coral *Favia speciosa* (Dana) around Singapore. *Hydrobiologia* **444**, 227–235 (2001).

[66] Januchowski-Hartley, S. R. et al. Small instream infrastructure: comparative methods and evidence of environmental and ecological responses. *Ecol. Solut. Evid.* **1**, e12026 (2020).

[67] Todd, P. A. Morphological plasticity in scleractinian corals. *Biol. Rev.* **83**, 315–337 (2008). 18979594 10.1111/j.1469-185x.2008.00045.x

[68] Hoeksema, B. W., Timmerman, R.F., Spaargaren, R., Smith-Moorhouse, A., van der Schoot, R.J, Langdorn-Down, S.J., Harper, C.E. Morphological modifications and injuries of corals caused by symbiotic feather duster worms (Sabellidae) in the Caribbean. *Diversity* **14**, 332 (2022).

[69] Weber, M., Lott, C. & Fabricius, K. E. Mechanisms of damage to corals exposed to sedimentation. *Proc. Natl. Acad. Sci. USA*. **109**, E1558–E1567 (2012). 22615403 10.1073/pnas.1100715109 PMC3386076

[70] Johnson, J. A. et al. Palaeoecological records of coral community development on a turbid, nearshore reef complex: baselines for assessing ecological change. *Coral Reefs*. **36**, 685–700 (2017). 32025194 10.1007/s00338-017-1561-1 PMC6979561

[71] Chan, Y. S. et al. Holocene coral assemblages reveal similarities to living communities in Singapore’s urban reef environment. *Palaeogeogr Palaeoclimatol Palaeoecol*. **669**, 112930 (2025).

[72] Hynes, M. G. et al. Impact of Holocene relative sea-level changes on patch reef-island development in the Spermonde Archipelago, South Sulawesi, Indonesia. *Holocene* (2025). 10.1177/09596836251313628

[73] Morgan, K. M., Perry, C. T., Arthur, R., Williams, H. T. P. & Smithers, S. G. Projections of coral cover and habitat change on turbid reefs under future sea-level rise. *Proc. R. Soc. B* 287, 20200541 (2020). 10.1098/rspb.2020.0541 PMC7329041 32546095

[74] Joppien, M. & Morgan, K. Benthic mud content is a strong indicator of coral cover and ecosystem recovery on turbid coral reefs. *Mar. Pollut Bull.* **212**, 117596 (2025). 39855066 10.1016/j.marpolbul.2025.117596

[75] Ryan, E. J., Smithers, S. G., Lewis, S. E., Clark, T. R. & Zhao, J. X. Chronostratigraphy of Bramston Reef reveals a long-term record of fringing reef growth under muddy conditions in the central Great Barrier Reef. *Palaeogeogr Palaeoclimatol Palaeoecol*. **441**, 734–747 (2016).

